# Plasma SIRT7 as a novel biomarker for coronary artery disease and rehospitalization risk in hypertensive patients: a cross-sectional and longitudinal study

**DOI:** 10.1007/s11739-025-04092-1

**Published:** 2025-08-30

**Authors:** Xinyu Zhou, Ying Liu, Ying Guo, Ning Wang, Siyuan Wang, Jiawei Song, Zhaojie Dong, Xiaoyan Yang, Yufei Chen, Jing Li, Lin Zhao, Ying Dong, Jiuchang Zhong

**Affiliations:** 1 Heart Center and Beijing Key Laboratory of Hypertension, Beijing Chaoyang Hospital, Capital Medical University, 8th Gongtinanlu Rd, Chaoyang District, Beijing, 100020 China; 2 Department of Cardiology, Beijing Chaoyang Hospital, Capital Medical University, Beijing, 100020 China; 3 Department of Geriatrics, Beijing Tongren Hospital, Capital Medical University, Beijing, 100005 China; 4 Medical Research Center, Beijing Chaoyang Hospital, Capital Medical University, Beijing, 10020 China

**Keywords:** SIRT7, Hypertension, Coronary artery disease, Biomarker, Rehospitalization

## Abstract

The exact role of SIRT7 in hypertensive patients with coronary artery disease (CAD) remains elusive. This study aimed to explore plasma SIRT7 levels and their clinical significance in this population. From July 2022 to June 2024, 222 hypertensive patients (106 with isolated hypertension and 116 with hypertension plus CAD) were enrolled. Plasma SIRT7 was measured by ELISA, and participants were followed for rehospitalization (median 804 days). ROC curves, LASSO regression, and Cox proportional hazards models were used for statistical analysis. Hypertensive CAD patients had significantly higher SIRT7 levels than those without CAD (*P* < 0.001). Plasma SIRT7 levels were positively correlated with CK (*R* = 0.36, *P* < 0.001) and CK-MB (*R* = 0.50, *P* < 0.001), and negatively with HDL-C (*R* = −0.23, *P* < 0.001). The optimal cutoff of SIRT7 for diagnosing CAD demonstrated 94.0% sensitivity and 75.5% specificity, exhibiting an AUC of 0.917, superior to CK, CK-MB, and HDL-C. Multivariate analysis identified SIRT7 as an independent CAD risk factor (adjusted OR = 1.97, 95% CI 1.48–2.63). Restricted cubic spline (RCS) analysis revealed a linear correlation between SIRT7 and rehospitalization (*P* for overall = 0.038), but no non-linear relationship (*P* for non-linear = 0.164). Elevated SIRT7 predicted rehospitalization risk (HR = 1.15, 95% CI 1.02–1.29) and improved risk prediction, with an integrated discrimination index (IDI) of 21.8% and net reclassification improvement (NRI) of 51.0%. Plasma SIRT7 acts as both a diagnostic marker for CAD and a predictor of rehospitalization in hypertensive patients, indicating its dual role in pathogenesis and clinical monitoring. Targeting SIRT7 signaling may offer a new therapeutic strategy for hypertension-associated CAD.

**Supplementary Information:**

The online version contains supplementary material available at 10.1007/s11739-025-04092-1.

## Introduction

Hypertension is the leading global health challenge due to its high prevalence and resulting various complications, such as cardiovascular diseases, chronic kidney diseases, stroke, and other health issues [1, 2]. Coronary artery disease (CAD) is considered a major risk factor affecting human health, which includes stable and unstable angina, myocardial infarction, or sudden cardiac death [3]. Hypertension and CAD are intimately related, with elevated blood pressure contributing to a higher incidence of CAD [4]. Notably, hypertension combined with CAD can significantly impair patients’ quality of life and impose a considerable burden on both families and society. Consequently, effective prevention and early detection of patients with hypertension and CAD are urgent.

Sirtuin7 (SIRT7) is a nicotinamide adenine dinucleotide (NAD)-dependent histone deacetylase, primarily located in the nucleolus, which has obtained growing attention for its regulatory role in cellular stress, genome stability, metabolic regulation, aging, and cancer [5]. It is worth noting that most previous studies regarding SIRT7 and cardiovascular diseases have been concentrated at the animal level. For instance, research has shown that SIRT7 exerts protective effects on cardiovascular health [6]. Specifically, SIRT7 inhibited the proliferation and migration of vascular smooth muscle cells, thus offering a novel therapeutic strategy against atherosclerosis [7]. However, under certain pathological conditions, SIRT7 has been implicated in disease progression; for example, under Ang-II stimulation, the overexpression of SIRT7 has been found to exacerbate fibrosis [8].

Recently, emerging evidence from our research group has begun to reveal that, compared with nonhypertensive patients, the plasma level of SIRT7 is lower in hypertensive patients [9]. However, the interplay between SIRT7 and the coexistence of hypertension with CAD remains poorly understood. Particularly, it remains unclear whether, under hypertensive conditions, SIRT7 may similarly exacerbate disease risk in humans as observed in certain pathological contexts in animal models. Therefore, the objective of this study was to explore the plasma SIRT7 expressions in hypertensive patients with or without CAD and to assess the correlation between SIRT7 and hospital readmissions during the follow-up period.

## Methods

### Study design and populations

The study was conducted at Beijing Tongren Hospital affiliated with Capital Medical University. The patients were recruited between July 2022 and June 2024. Based on the 2020 guidelines of the International Society of Hypertension, hypertension was defined as having a systolic blood pressure (SBP) ≥ 140 mm Hg, and/or diastolic blood pressure (DBP) of ≥ 90 mm Hg, or the use of antihypertensive medication within the past 2 weeks [10]. CAD was diagnosed based on angina pectoris manifestations, alterations in the electrocardiogram, and coronary angiography, which indicated that the major vessel had a degree of stenosis greater than or equal to 50% [11]. Furthermore, patients suffering from severe hepatic and renal insufficiency, autoimmune disease, thyroid dysfunction, pregnancy, complicated acute infection, hematologic conditions, and active malignant tumors were excluded from this study. The enrolled patients were categorized into two groups: the hypertension-only group (HTN), comprising individuals with a clinical diagnosis of hypertension but no evidence of CAD, and the hypertension with CAD group (HTN_CAD), which included patients diagnosed with both hypertension and CAD based on the diagnostic criteria described above. After careful consideration of the relevant parameters, our calculation indicates that group sample sizes of 106 in the HTN group and 116 in the HTN_CAD group can achieve a power of 87.531% to detect an odds ratio of the group proportions of 2.430. Ethics approval was obtained from the Ethics Committee of Beijing Tongren Hospital (No. TRECKY 2021-172) and adhered to the principles outlined in the Declaration of Helsinki. Informed consent was obtained from all participants prior to the investigation.

### Data collection

Comprehensive medical records were utilized to obtain demographic information, including age, gender, tobacco and alcohol consumption, past medical history, as well as details of their ongoing medical therapy. Venous blood samples were collected at least 8 h after the last meal. The blood routine examination, encompassing white blood cell count (WBC), neutrophilic granulocyte percentage (NE%), and hemoglobin (Hb), was performed using complete blood count. The serum levels of high-sensitivity C-reactive protein (hs-CRP), fasting blood glucose (FBG), alanine aminotransferase (ALT), aspartate aminotransferase (AST), creatine kinase (CK), MB isoenzyme of creatine kinase (CK-MB), low-density lipoprotein cholesterol (LDL-C), high-density lipoprotein cholesterol (HDL-C), total cholesterol (TC), triglyceride (TG), lipoprotein(a) (Lp(a)), creatinine (Cr), blood urea nitrogen (BUN), uric acid (UA) and D-dimer were examined using an automatic biochemical analyzer (AU5821, Beckman Coulter, USA) according to standard protocols. Plasma glycated hemoglobin (HbA1c) was determined by automatic glycohemoglobin analyzer (HCL723G8, SYSMEX, Japan).

### SIRT7 enzyme immunoassay

Detailed methods used in the survey have been described previously [9]. Plasma was obtained from the peripheral vein by centrifugation at 3000 revolutions per minute (rpm) at 4 °C for 10 min (min) and collecting plasma supernatant. Plasma levels of SIRT7 were quantified using the enzyme-linked immunosorbent assay (ELISA) sandwich method, according to the manufacturer’s instructions. Spectrophotometric measurements were conducted using the Thermo Scientific Multiscan GO ELISA reader (Finland), with an excitation wavelength set at 450 nm.

### Endpoints and follow-up

Follow-up interviews were systematically conducted via structured telephone calls, correspondence, and scheduled outpatient visits by trained staff, with surveillance continuing until December 2024 (median follow-up duration: 804 days; interquartile ranges: 650–1050 days). The primary outcome was all-cause rehospitalization, defined as the number of study participants hospitalized for any reason [12].

### Statistical analysis

Data analyses were conducted using SPSS (version 25; IBM Corp., Armonk, NY, USA), R software (version 4.3.1; R Foundation for Statistical Computing, Vienna, Austria) and PASS software (Version 15.0, NCSS, LLC. Kaysville, Utah, USA). Continuous variables were expressed as mean ± standard deviation (SD) when normally distributed or median with interquartile range (IQR) when non-normally distributed, while categorical variables were presented as numbers and percentages. Student’s t-test or Mann–Whitney U test was used for continuous variables, and Chi-squared test was performed for categorical variables. The relationship between plasma SIRT7 levels and clinical features was evaluated using Spearman’s rank correlation analysis. Furthermore, the areas under the receiver operating characteristic (ROC) curves (AUCs) for SIRT7 and traditional CAD risk factors—especially those showing significant correlation with SIRT7—were compared to evaluate their reliability as predictive markers of CAD. Statistical comparisons of the ROC curves were performed using DeLong’s test [13] with MedCalc statistical software (version 11.4.2.0; MedCalc Software, Ostend, Belgium).

For cross-sectional study, potential predictors showing significant associations (*P* < 0.05) in univariate analyses were retained for subsequently modeling. These variables were then subjected to least absolute shrinkage and selection operator (LASSO) regression analysis, a method renowned for its effectiveness in addressing issues of multicollinearity and overfitting often caused by an overabundance of predictors [13]. In terms of model selection, K-fold cross-validation is highly recommended over bootstrap [14]. We utilized tenfold cross-validation, which could standardize and centralize included variables, to determine the optimal lambda value. A model exhibiting good performance with minimal independent variables was constructed using “lambda + 1se” [15]. Subsequently, a prediction model was detected by multivariable stepwise logistic regression analysis.

For the longitudinal analysis, we assessed the overall and nonlinear relationship between continuous SIRT7 and rehospitalization using the restricted cubic spline analysis (RCS), and the reference point was set to the optimal cut-off point. Subsequently, participants were dichotomized into high- and low-SIRT7 groups based on this predefined cut-off point. Kaplan–Meier curve was plotted to compare rehospitalization-free survival probabilities between the two groups, with statistical significance determined by the log-rank test. Cox proportional regression analysis was utilized to identify potential risk factors for incident rehospitalization, with results expressed as hazard ratios (HRs) and 95% confidence intervals (CIs). The proportional hazards assumption was rigorously assessed by a Kolmogorov-type supremum test on the basis of cumulative sums of Martingale-based residuals over follow-up times and covariate variables. First, univariate Cox regression analysis was performed to assess the association between each predictor variable and the occurrence of rehospitalization. Variables showing statistically significant associations (*P* < 0.05) in the univariate analysis were then included in a stepwise multivariate Cox regression model to determine independent predictors of rehospitalization. Additionally, the net reclassification improvement (NRI) and integrated discrimination index (IDI) indices were calculated to estimate the predictive value of SIRT7 beyond conventional risk factors. A two-sided *P*-value < 0.05 was considered statistically significant.

## Results

### Baseline characteristics of study population

A total of 222 subjects with hypertension were recruited, comprising 106 individuals (47.7%) with a clinical diagnosis of hypertension and 116 individuals (52.3%) diagnosed with hypertension in conjunction with CAD. The baseline characteristics of the study participants are shown in Table 1 (mean age: 65.5 years, males: 68.9%). Obviously, in comparison to the hypertensive group, patients with hypertension and CAD exhibited a significantly higher level of SIRT7 (*P* < 0.001). Individuals afflicted concurrently with hypertension and CAD were predominantly male, with a higher prevalence of smoking history, hyperlipidemia, and antiplatelet agent usage. In addition, participants with both hypertension and CAD had elevated heart rates along with a marked increase in blood biochemical markers including WBC, NE%, plasma concentrations of ALT, AST, CK, CK-MB, and hs-CRP, as well as decreased HDL-C concentration compared to those with hypertension alone.

**Table 1:** The clinical baseline characteristics and laboratory data between hypertensive patients with (HTN_CAD) or without CAD (HTN)

Variable	Total (*n* = 222)		HTN (*n* = 106)	HTN_CAD (*n* = 116)	*P*-value
Demographic					
Age, years	65.5 ± 7.5		66.3 ± 7.6	64.8 ± 7.4	0.141
Male	153 (68.9%)		66 (62.3%)	87 (75.0%)	0.041
BMI, kg/m^2^	25.6 ± 3.1		25.8 ± 3.2	25.3 ± 3.0	0.234
Drinking	68 (30.6%)		29 (27.4%)	39 (33.6%)	0.312
Smoking	72 (32.4%)		17 (16.0%)	55 (47.4%)	< 0.001
SBP, mmHg	137.4 ± 16.5		137.5 ± 15.3	137.4 ± 17.5	0.950
DBP, mmHg	78.4 ± 11.1		77.8 ± 10.2	78.9 ± 11.9	0.450
HR, bpm	72.5 (65.0, 82.0)		71.0 (63.8, 77.3)	78.0 (66.0, 86.8)	< 0.001
Comorbidities					
T2DM	121 (54.5%)		56 (52.8%)	65 (56.0%)	0.632
Hyperlipidemia	184 (82.9%)		77 (72.6%)	107 (92.2%)	< 0.001
Laboratory data					
WBC, × 10^9^/L	6.8 (5.4, 8.6)		5.9 (5.1, 7.0)	8.2 (6.0, 10.0)	0.005
NE%	63.4 ± 13.1		57.6 ± 9.1	68.7 ± 13.9	< 0.001
Hb, g/L	136.0 (124.0, 144.0)		138.0 (129.0, 145.0)	133.0 (119.0, 144.0)	< 0.001
HbA1c, %	6.4 (5.8, 7.0)		6.5 (5.8, 6.8)	6.4 (5.9, 7.4)	0.174
FBG, mmol/L	6.7 ± 2.5		6.6 ± 2.2	6.7 ± 2.7	0.767
ALT, U/L	21.0 (14.0, 31.3)		17.0 (13.0, 25.8)	23.5 (17.0, 36.5)	< 0.001
AST, U/L	21.0 (17.0, 31.0)		20.0 (17.0, 23.3)	27.0 (18.0, 55.9)	< 0.001
CK, U/L	94.0 (62.0, 171.3)		75.0 (57.0, 105.3)	126.5 (69.0, 379.5)	< 0.001
CK-MB, U/L	13.0 (9.0, 83.8)		10.0 (8.8, 13.0)	75.5 (14.0, 194.5)	0.001
Lp(a), mg/dL	12.2 (6.1, 29.9)		9.5 (4.6, 26.3)	14.9 (7.4, 35.0)	0.223
TG, mmol/L	1.2 (0.9, 1.8)		1.3 (0.9, 1.8)	1.1 (0.9, 1.8)	0.310
TC, mmol/L	4.1 ± 1.1		4.3 ± 1.2	4.0 ± 1.0	0.044
LDL-C, mmol/L		2.5 ± 0.9	2.4 ± 0.9	2.5 ± 0.9	0.481
HDL-C, mmol/L		1.1 ± 0.4	1.3 ± 0.5	1.0 ± 0.3	< 0.001
D-dimer, mg/L		0.3 (0.2, 0.6)	0.3 (0.2, 0.5)	0.3 (0.2, 0.8)	< 0.001
UA, μmol/L		332.3 ± 90.3	343.8 ± 86.5	321.9 ± 92.9	0.072
BUN, mmol/L	5.5 (4.6, 6.8)		5.3 (4.3, 6.5)	5.8 (4.7, 7.0)	0.010
hs-CRP, mg/L	1.4 (0.4, 5.6)		0.8 (0.4, 2.2)	2.6 (0.6, 11.4)	< 0.001
Cr, μmol/L	69.6 ± 12.8		69.3 ± 11.8	69.9 ± 13.7	0.749
SIRT7, ng/mL	2.8 (1.0, 5.6)		1.0 (0.6, 2.1)	5.4 (3.2, 6.7)	< 0.001
Medical therapy					
Antiplatelet	153 (68.9%)		42 (36.2%)	111 (95.7%)	< 0.001
OAD	99 (44.6%)		49 (46.2%)	50 (43.1%)	0.640

Data are expressed as mean ± SD, median (interquartile range), or percentages in parentheses

*HTN* hypertension, *CAD* coronary artery disease, *BMI* body mass index, *SBP* systolic blood pressure, *DBP* diastolic blood pressure, *HR* heart rate, *T2DM* type 2 diabetes mellitus, *WBC* white blood count, *NE%* neutrophilic granulocyte percentage, *Hb* hemoglobin, *HbA1c* Hemoglobin A1c, *FBG* fasting blood glucose, *ALT* alanine aminotransferase, *AST* aspartate aminotransferase, *CK* creatine kinase, *CK-MB* MB isoenzyme of creatine kinase, *Lp(a)* lipoprotein(a), *TG* triglyceride, *TC* total cholesterol, *LDL-C* low-density lipoprotein cholesterol, *HDL-C* high-density lipoprotein cholesterol, *SIRT7* sirtuin 7, *UA* uric acid, *BUN* blood urea nitrogen, *hs-CRP* high-sensitivity C-reactive protein, *Cr* creatinine, *OAD* oral antidiabetic drug

### Correlation between plasma SIRT7 and clinical indicators

Subsequently, we investigated the relationship between SIRT7 and six conventional risk factors for CAD in hypertensive patients, which included lipid profile parameters (TC, TG, HDL-C and LDL-C) and cardiac enzymes (CK and CK-MB). Remarkably, as depicted in Figure S1, both CK (*R* = 0.36, *P* < 0.001, Figure S1A) and CK-MB (*R* = 0.5, *P* < 0.001, Figure S1B) revealed a positive correlation with SIRT7 plasma levels. In contrast, HDL-C (*R* = −0.23, *P* < 0.001, Figure S1F) demonstrated a negative correlation with plasma SIRT7 levels.

### Plasma SIRT7 levels were qualified in distinguishing CAD among hypertensive patients

ROC curve analysis indicated that the optimal cutoff threshold for plasma SIRT7 levels in identifying hypertensive patients with concomitant CAD was established at 2.14 ng/mL, achieving a high sensitivity of 94.0% and a specificity of 75.5%, underscoring its potential diagnostic utility. In addition, a comparative assessment of the diagnostic efficacies of SIRT7, CK, CK-MB, and HDL-C—the indicators that demonstrated a significant correlation with SIRT7 as shown in Figure S1—was carried out. As illustrated in Figure S2, SIRT7 exhibited superior diagnostic accuracy (AUC: 0.917, 95% CI 0.882–0.951) for distinguishing hypertension in conjunction with CAD compared to CK (AUC: 0.698, 95% CI 0.629–0.768 *P* < 0.001), CK-MB (AUC: 0.814, 95% CI 0.750–0.879 *P* < 0.001) and HDL-C (AUC: 0.674, 95% CI 0.604–0.745 *P* < 0.001).

### SIRT7 is an independent risk factor of CAD among hypertensive patients

Initially, a univariate logistic regression analysis was performed to evaluate the individual effects of variables on CAD among hypertensive patients (Table S1). Univariate logistic regression analysis revealed that factors including male gender, history of smoking, comorbidity of hyperlipidemia, elevated heart rate, high levels of SIRT7, WBC, NE%, hemoglobin, ALT, AST, CK, CK-MB, TC, BUN, hs-CRP, as well as use of antiplatelet agents, were significantly associated with an increase in the risk of CAD among hypertensive patients. However, elevated levels of HDL-C were found to decrease the risk of CAD.

**Fig. 1:**
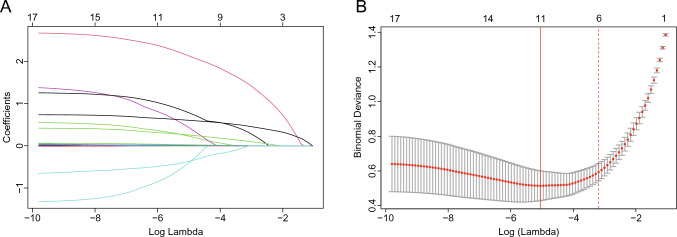
Variable selection using LASSO regression analysis. **A** LASSO coefficient profiles of the 17 variables. A coefficient profile plot was produced against the log (lambda) sequence. **B** A tenfold cross-validation was used in the LASSO regression. Binomial deviance was plotted versus log (lambda). Dotted vertical lines were drawn at the optimal values by utilizing the minimum criteria (left full line) and the 1 standard error criterion (right dotted line)

Additionally, we performed the LASSO regression model on all relevant characteristic variables that were identified as statistically significant features in the univariate analysis (Table S1). Among the 17 pertinent characteristic variables, 6 potential predictors were selected through the LASSO regression model (Fig. 1A, B). Finally, four independent predictors of CAD among hypertensive patients were identified by multivariate stepwise logistic regression analysis (Table 2). Notably, after adjustment for WBC, CK-MB levels, and antiplatelet therapy use, each unit increase in SIRT7 was associated with a 97% elevated risk of CAD (adjusted OR: 1.97; 95% CI 1.48–2.63; *P* < 0.001).

**Table 2:** Predictors for the risk of CAD among hypertensive patients through multivariate stepwise logistic regression analysis

Variable	*β*	OR	95% CI	*P* value
WBC, × 10^9^/L	0.49	1.63	1.17–2.27	0.004
CK-MB, U/L	0.05	1.05	1.02–1.09	0.004
Antiplatelet	3.00	20.13	3.26–124.30	0.001
SIRT7, ng/mL	0.68	1.97	1.48–2.63	< 0.001

Other abbreviations are shown in Table 1

*OR* odds ratio, *95% CI* 95% confidence interval

### Association of SIRT7 and rehospitalization

**Fig. 2:**
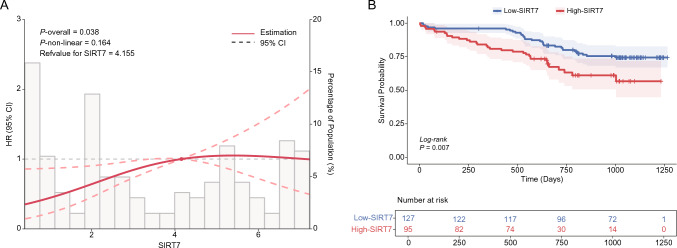
Association between SIRT7 expression levels and rehospitalization during follow-up. **A** Dose–response relationship of SIRT7 with HR for rehospitalization, analyzed using restricted cubic splines. The adjusted HRs (red lines) and 95% confidence intervals (dashed lines). Overall *P*-value = 0.038, non-linear *P*-value = 0.164. Reference SIRT7 value = 4.155 ng/mL. **B** Kaplan–Meier survival curves comparing survival probability in patients grouped by Low- and High-SIRT7 expression. The log-rank test showed a significant survival difference (*P* = 0.007). Number at risk at each time point is shown in the table below the plot

During the follow-up period, a total of 64 patients experienced rehospitalization. The results of the RCS analysis indicated a correlation between SIRT7 and rehospitalization (*P* for overall = 0.038), but not a non-linear relationship (*P* for non-linear = 0.164) (Fig. 2A). Through detailed analysis of the RCS plot, the optimal cutoff value for SIRT7 expression was determined to be 4.155. This threshold has been used to stratify patients into high (≥ 4.155) and low (< 4.155) SIRT7 expression groups for the Kaplan–Meier survival analysis. The Kaplan–Meier curve demonstrated a significant difference in survival probability between high SIRT7 and low SIRT7 groups (log-rank *P* = 0.007, Fig. 2B). As shown in Table 3, univariate Cox regression analysis revealed that higher SIRT7 levels (HR = 1.23, 95% CI 1.11–1.37, *P* < 0.001), along with conventional risk factors including type 2 diabetes mellitus (HR = 2.05, 95% CI 1.21–3.27, *P* = 0.008), AST (HR = 1.31, 95% CI 1.14–1.50, *P* < 0.001) and CK-MB (HR = 1.48, 95% CI 1.25–1.75, *P* < 0.001) were significantly associated with rehospitalization. In addition, even after accounting for established risk factors (univariate analysis *P* < 0.05), plasma SIRT7 retained a significant association with rehospitalization (HR = 1.15, 95% CI 1.02–1.29, *P* = 0.022), suggesting its potential as an independent predictor for rehospitalization. Strikingly, SIRT7 levels substantially improved rehospitalization prediction over the conventional risk factors, with robust evidence from both discrimination (IDI of 21.8%, *P* < 0.001) and reclassification (NRI of 51.0%, *P* < 0.001) metrics.

**Table 3:** Univariate and multivariate Cox analysis for prediction of the all-cause hospital readmission

Variables	Univariate analyses		Multivariate analyses	
	HR (95% CI)	*P* value	HR (95% CI)	*P* value
SIRT7, ng/mL	1.23 (1.11–1.37)	< 0.001	1.15 (1.02–1.29)	0.022
Age, years	1.00 (0.97–1.03)	0.969		
Male	1.75 (0.97–3.17)	0.065		
BMI, kg/m^2^	0.98 (0.90–1.06)	0.636		
Drinking	0.88 (0.51–1.52)	0.649		
Smoking	1.30 (0.78–2.18)	0.310		
SBP, mmHg	1.14 (0.89–1.47)	0.293		
DBP, mmHg	1.18 (0.92–1.51)	0.191		
HR, bpm	1.17 (0.92–1.48)	0.209		
T2DM	2.05 (1.21–3.47)	0.008	1.99 (1.17–3.37)	0.011
Hyperlipidemia	2.01 (0.92–4.42)	0.082		
WBC, × 10^9^/L	1.27 (1.03–1.56)	0.028		
NE%	1.28 (0.97–1.68)	0.078		
Hb, g/L	0.89 (0.72–1.10)	0.276		
HbA1c, %	1.11 (0.88–1.40)	0.392		
FBG, mmol/L	1.31 (1.06–1.61)	0.012		
ALT, U/L	1.19 (0.97–1.47)	0.090		
AST, U/L	1.31 (1.14–1.50)	< 0.001	1.20 (1.01–1.43)	0.036
CK, U/L	1.31 (1.10–1.57)	0.003		
CK-MB, U/L	1.48 (1.25–1.75)	< 0.001	1.28 (1.04–1.57)	0.021
LP(a), mg/dL	0.92 (0.70–1.21)	0.539		
TG, mmol/L	0.89 (0.68–1.16)	0.383		
TC, mmol/L	0.88 (0.69–1.14)	0.335		
LDL-C, mmol/L	0.98 (0.76–1.25)	0.844		
HDL-C, mmol/L	0.83 (0.63–1.11)	0.205		
D-dimer, mg/L	1.08 (0.91–1.29)	0.368		
UA, μmol/L	1.22 (0.95–1.56)	0.127		
BUN, mmol/L	1.17 (0.92–1.49)	0.194		
hs-CRP, mg/L	0.89 (0.60–1.33)	0.567		
Cr, μmol/L	1.14 (0.90–1.46)	0.276		
Antiplatelet	2.33 (1.26–4.30)	0.007		
OAD	1.63 (1.00–2.67)	0.051		

Other abbreviations are shown in Table 1

*HR* hazard ratio, *95% CI* 95% confidence interval

## Discussion

This study provides the first investigation into the association between SIRT7 and CAD in hypertensive patients. Initially, through a cross-sectional analysis, SIRT7 levels are significantly elevated in hypertensive patients with CAD compared to those without CAD. Additionally, we observed a positive correlation between SIRT7 and both CK and CK-MB, while there is an inverse relationship with HDL-C. Notably, SIRT7 remains an independent risk factor for CAD among hypertensive patients even after rigorous statistical adjustments. Subsequently, we conducted a follow-up of these participants to examine the relationship between baseline plasma SIRT7 levels and rehospitalization, revealing that SIRT7 is an independent predictor of rehospitalization in hypertensive patients. More importantly, incorporating elevated SIRT7 levels into conventional risk assessment models enhances their predictive value for rehospitalization. Collectively, these findings suggest that SIRT7 not only increases the risk of CAD but also serves as a potential biomarker for monitoring rehospitalization risk among hypertensive patients.

The mammalian genome encodes seven members of the SIRT family, which is a group of deacetylases whose activity is highly dependent on intracellular NAD^+^ levels. SIRT7, the latest characterized SIRT, is localized in the nucleus and serves as a pivotal regulator in various biological processes, including ribosomal RNA synthesis, DNA damage repair, aging, metabolism, oxidative stress responses, cell proliferation, and tumorigenesis [16]. Previous studies have demonstrated that SIRT7 expression varies across different disease conditions. Within the realm of tumor-associated disorders, elevated levels of SIRT7 are linked to poor survival outcomes in patients diagnosed with hepatocellular carcinoma [17]. Furthermore, research on respiratory diseases has shown that SIRT7, which is upregulated in airway epithelial cells from asthma patients, promotes airway remodeling by modulating TGF-β1-induced proliferation and migration of airway smooth muscle cells [18, 19].

In cardiovascular disease, SIRT7 exhibits a dual regulatory role. Zhang et al. [7] demonstrated that SIRT7 suppresses vascular smooth muscle cell proliferation and migration via activation of the Wnt/β-catenin signaling pathway, suggesting its therapeutic potential for atherosclerosis. In parallel, emerging evidence indicates that SIRT7 overexpression attenuates endothelial cell inflammatory responses by regulating KLF4 deacetylation and ameliorates pulmonary hypertension [20]. Conversely, during fibrosis, Ang-II stimulated upregulation of SIRT7 expression and phosphorylation levels in cardiac fibroblasts, promoting the transformation of cardiac fibroblasts to myofibroblasts, and overexpression of SIRT7 exacerbated Ang-II-induced fibrosis [8]. Similarly, with previous findings, analysis of our cross-sectional cohort demonstrated markedly elevated SIRT7 expression levels in hypertensive patients diagnosed with CAD.

Given that CAD is widely recognized as a consequence of chronic low-grade vascular inflammation [21, 22], and hypertension commonly coexists with CAD, exacerbating vascular inflammation [23]. The upregulated expression of SIRT7 in hypertensive patients complicated by CAD might be attributed to its pro-inflammatory properties, which primarily modulate inflammatory responses via the NF-κB signaling pathway—a pivotal regulator of inflammation. Chronic elevation of pro-inflammatory cytokines (e.g., IL-1β, IL-6, TNF-α) potently stimulates rapid translocation of NF-κB from the cytoplasm to the nucleus [24]. SIRT7 might facilitate this process, promoting NF-κB activation. Previous studies utilizing murine models have demonstrated that SIRT7 deletion mitigates inflammation through suppression of NF-κB signaling pathways, evidenced by reduced nuclear phosphorylation of p65 and decreased pro-inflammatory mediator mRNA levels [25]. Additionally, elevated SIRT7 levels are implicated in inflammatory bowel disease, while inhibiting SIRT7 ameliorates disease progression by reducing inflammatory factor expression, further validating its pro-inflammatory role [26]. Thus, in patients suffering from hypertension concurrent with CAD, the body’s response to persistent inflammation might trigger a compensatory increase in SIRT7 expression. This, in turn, has the potential to initiate a self-perpetuating inflammatory cycle that further accelerates the advancement of both hypertension and CAD. Notably, a key finding of our study is that elevated SIRT7 expression was independently associated with an increased risk of rehospitalization during follow-up. The current animal researches on SIRT7 are predominantly short-term observations, and its precise mechanisms affecting long-term disease outcomes require further in-depth investigation.

Our study has several limitations that should be considered. First, as a single-center investigation with a moderate sample size, our findings require validation in larger, multi-center cohorts to confirm their generalizability. Second, while we established a diagnostic cutoff for SIRT7 (2.14 ng/mL) through ROC statistical analysis, this threshold needs independent verification in different patient populations. Third, the cross-sectional design limits our ability to determine whether SIRT7 elevation contributes to or results from CAD progression in hypertensive patients. Additionally, the underlying molecular mechanisms linking SIRT7 to long-term disease progression remain incompletely understood and need further mechanistic exploration. These aspects should be addressed in future longitudinal and experimental studies before considering clinical application of SIRT7 as a diagnostic biomarker.

## Conclusion

In summary, plasma SIRT7 levels are significantly elevated in hypertensive patients with CAD compared to those with isolated hypertension. Multivariate analysis confirms elevated SIRT7 as an independent risk factor for hypertension comorbid with CAD. More importantly, SIRT7 is identified as an independent predictor of rehospitalization in hypertensive patients, and its inclusion in traditional risk assessment models improves the prediction of rehospitalization. Clinically, these findings highlight SIRT7 as both a contributor to CAD development and a valuable biomarker for assessing rehospitalization risk in hypertensive patients. Targeting the SIRT7 signaling pathway presents a promising new strategy for preventing and treating hypertension complicated by CAD, offering potential for more effective patient management and improved clinical outcomes.

## Supplementary Information

Below is the link to the electronic supplementary material.

Supplementary file1 Figure S1. SIRT7 and its correlation with major risk factors of CAD in hypertensive patients. **A** The association between SIRT7 and CK; **B** The association between SIRT7 and CK-MB; **C** The association between SIRT7 and TG; **D** The association between SIRT7 and TC; **E** The association between SIRT7 and LDL-C; **F** The association between SIRT7 and HDL-C. CAD, coronary artery disease; SIRT7, sirtuin 7; CK, creatine kinase; CK-MB, MB isoenzyme of creatine kinase; TG, triglyceride; TC, total cholesterol; LDL-C, low-density lipoprotein cholesterol; HDL-C, high-density lipoprotein cholesterol (PDF 1210 KB)

Supplementary file2 Figure S2. Diagnostic performance of SIRT7, CK, CK-MB, and HDL-C in hypertensive patients with and without CAD: a receiver operating characteristic (ROC) analysis. **A** ROC curve analysis comparing the diagnostic performance of SIRT7 versus conventional biomarkers (CK, CK-MB, HDL-C) in hypertensive patients with CAD. SIRT7 showed superior discriminative ability, as reflected by its higher AUC value (AUC = 0.917, 95%CI: 0.882-0.951). CAD, coronary artery disease; SIRT7, sirtuin 7; CK, creatine kinase; CK-MB, MB isoenzyme of creatine kinase; HDL-C, high-density lipoprotein cholesterol (PDF 110 KB)

Supplementary file3 (DOCX 22 KB)

## Data Availability

The datasets that were used and/or analyzed during this study can be obtained from the corresponding author upon reasonable request.
