# Supplementary figures and images for: Plasma SIRT7 as a novel biomarker for coronary artery disease and rehospitalization risk in hypertensive patients: a cross-sectional and longitudinal study
Source: Intern Emerg Med. 2025 Aug 30;20(8):2369–77. doi: 10.1007/s11739-025-04092-1 (PMC12672676; doi:10.1007/s11739-025-04092-1)

**A**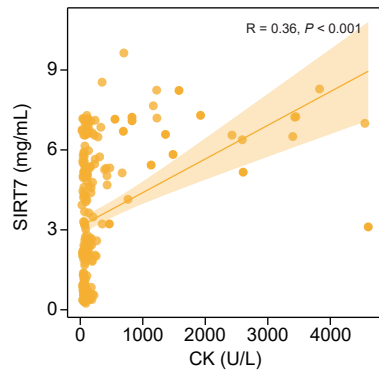**B**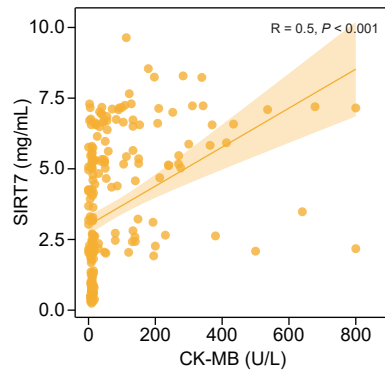**C**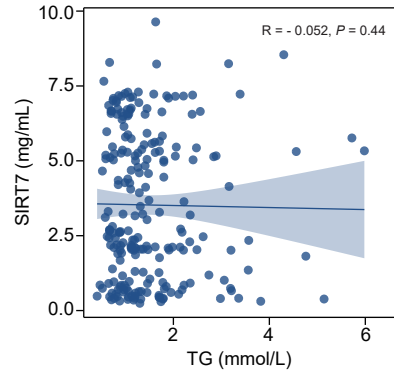**D**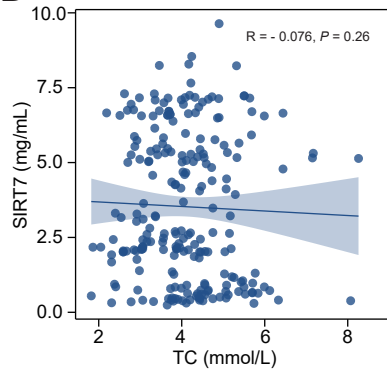**E**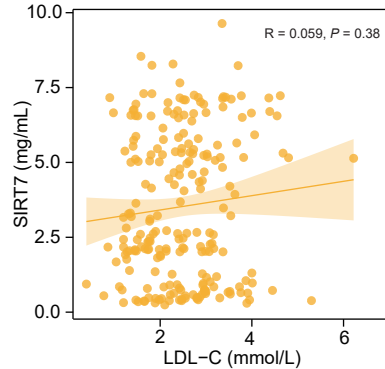**F**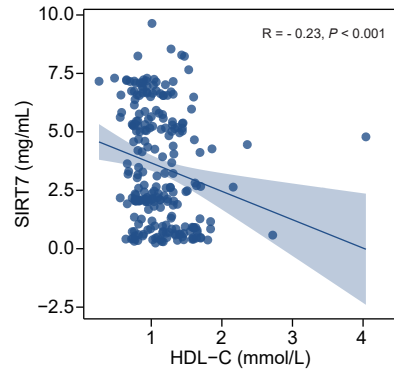

Supplement: Supplementary file 1 — Supplementary file1 Figure S1. SIRT7 and its correlation with major risk factors of CAD in hypertensive patients. A The association between SIRT7 and CK; B The association between SIRT7 and CK-MB; C The association between SIRT7 and TG; D The association between SIRT7 and TC; E The association between SIRT7 and LDL-C; F The association between SIRT7 and HDL-C. CAD, coronary artery disease; SIRT7, sirtuin 7; CK, creatine kinase; CK-MB, MB isoenzyme of creatine kinase; TG, triglyceride; TC, total cholesterol; LDL-C, low-density lipoprotein cholesterol; HDL-C, high-density lipoprotein cholesterol (PDF 1210 KB) [file 11739_2025_4092_MOESM1_ESM.pdf]

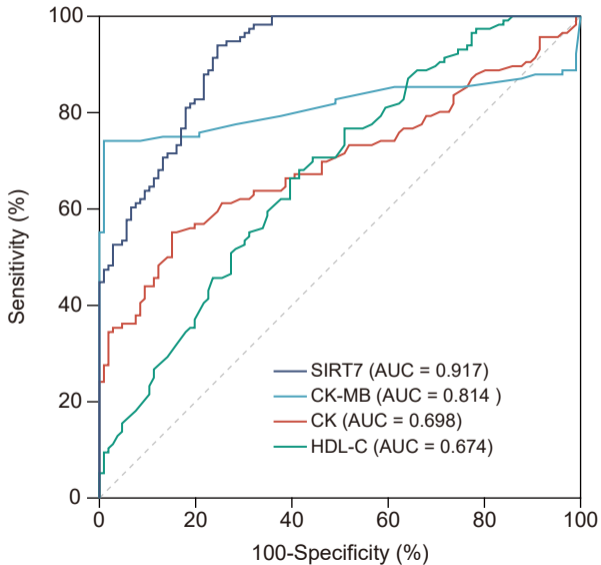

Supplement: Supplementary file 2 — Supplementary file2 Figure S2. Diagnostic performance of SIRT7, CK, CK-MB, and HDL-C in hypertensive patients with and without CAD: a receiver operating characteristic (ROC) analysis. A ROC curve analysis comparing the diagnostic performance of SIRT7 versus conventional biomarkers (CK, CK-MB, HDL-C) in hypertensive patients with CAD. SIRT7 showed superior discriminative ability, as reflected by its higher AUC value (AUC = 0.917, 95%CI: 0.882-0.951). CAD, coronary artery disease; SIRT7, sirtuin 7; CK, creatine kinase; CK-MB, MB isoenzyme of creatine kinase; HDL-C, high-density lipoprotein cholesterol (PDF 110 KB) [file 11739_2025_4092_MOESM2_ESM.pdf]
