# Supplementary material for: Plasma SIRT7 as a novel biomarker for coronary artery disease and rehospitalization risk in hypertensive patients: a cross-sectional and longitudinal study
Source: Intern Emerg Med. 2025 Aug 30;20(8):2369–77. doi: 10.1007/s11739-025-04092-1 (PMC12672676; doi:10.1007/s11739-025-04092-1)
Supplement: Supplementary file 3 — Supplementary file3 (DOCX 22 KB) [file 11739_2025_4092_MOESM3_ESM.docx]

| Table S1. Univariate logistic analysis on risk factors of HTN with CAD | | | | | |
| --- | --- | --- | --- | --- | --- |
| Variable | *β* | OR | 95% CI | *P* value |  |
| SIRT7, ng/mL | 0.89 | 2.43 | 1.97-3.00 | <0.001 |  |
| Age, years | -0.02 | 0.97 | 0.94-1.01 | 0.141 |  |
| Male | 0.60 | 1.82 | 1.02-3.23 | 0.042 |  |
| BMI, kg/m^2^ | -0.05 | 0.95 | 0.87-1.03 | 0.234 |  |
| Drinking | 0.30 | 1.35 | 0.76-2.39 | 0.313 |  |
| Smoking | 1.55 | 4.72 | 2.50-8.90 | <0.001 |  |
| SBP, mmHg | -0.0005 | 1.00 | 0.98-1.02 | 0.950 |  |
| DBP, mmHg | 0.009 | 1.01 | 0.99-1.03 | 0.452 |  |
| HR, bpm | 0.04 | 1.04 | 1.02-1.06 | <0.001 |  |
| T2DM | 0.13 | 1.14 | 0.67-1.93 | 0.632 |  |
| Hyperlipidemia | 1.50 | 4.48 | 2.01-10.00 | <0.001 |  |
| WBC, ×10^9^/L | 0.51 | 1.66 | 1.41-1.96 | <0.001 |  |
| NE% | 0.09 | 1.09 | 1.06-1.12 | <0.001 |  |
| Hb, g/L | -0.02 | 0.97 | 0.6-0.99 | 0.001 |  |
| HbA1c, % | 0.04 | 1.05 | 0.87-1.26 | 0.637 |  |
| FBG, mmol/L | 0.02 | 1.02 | 0.92-1.13 | 0.770 |  |
| ALT, U/L | 0.03 | 1.04 | 1.01-1.06 | 0.001 |  |
| AST, U/L | 0.05 | 1.06 | 1.03-1.08 | <0.001 |  |
| CK, U/L | 0.01 | 1.01 | 1.00-1.01 | <0.001 |  |
| CK-MB, U/L | 0.07 | 1.08 | 1.04-1.11 | <0.001 |  |
| LP(a), mg/dL | 0.01 | 1.01 | 1.00-1.02 | 0.069 |  |
| TG, mmol/L | -0.12 | 0.88 | 0.66-1.18 | 0.401 |  |
| TC, mmol/L | -0.26 | 0.77 | 0.60-0.99 | 0.044 |  |
| LDL-C, mmol/L | 0.10 | 1.11 | 0.83-1.48 | 0.480 |  |
| HDL-C, mmol/L | -2.15 | 0.12 | 0.05-0.29 | <0.001 |  |
| D-dimer, mg/L | 0.37 | 1.44 | 1.00-2.09 | 0.054 |  |
| UA, μmol/L | -0.002 | 1.00 | 0.99-1.00 | 0.073 |  |
| BUN, mmol/L | 0.25 | 1.29 | 1.08-1.53 | 0.006 |  |
| hs-CRP, mg/L | 0.11 | 1.11 | 1.04-1.18 | 0.001 |  |
| Cr, μmol/L | 0.003 | 1.00 | 0.98-1.02 | 0.748 |  |
| Antiplatelet | 3.52 | 33.82 | 12.73-89.84 | <0.001 |  |
| OAD | -0.12 | 0.88 | 0.52-1.50 | 0.640 |  |

OR, odds ratio; 95% CI, 95% confidence interval. Other abbreviations are shown in Table 1.
